# Inequality in mortality by occupation related to economic crisis from 1980 to 2010 among working-age Japanese males

**DOI:** 10.1038/srep22255

**Published:** 2016-03-03

**Authors:** Koji Wada, Stuart Gilmour

**Affiliations:** 1 Medical Officer, International Health Cooperation, National Center for Global Health and Medicine, 1-21-1 Toyama, Shinjuku-ku, Tokyo, 162-8655, Japan; 2 Assistant Professor, Department of Global Health Policy, Graduate School of Medicine, The University of Tokyo, 1-3-7 Hongo Bunkyo-ku Tokyo, 113-0033, Japan

## Abstract

The mortality rate for Japanese males aged 30–59 years in managerial and professional spiked in 2000 and remains worse than that of other occupations possibly associated with the economic downturn of the 1990s and the global economic stagnation after 2008. The present study aimed to assess temporal occupation-specific mortality trends from 1980 to 2010 for Japanese males aged 30–59 years for major causes of death. We obtained data from the Occupation-specific Vital Statistics. We calculated age-standardized mortality rates for the four leading causes of death (all cancers, suicide, ischaemic heart disease, and cerebrovascular disease). We used a generalized estimating equation model to determine specific effects of the economic downturn after 2000. The age-standardized mortality rate for the total working-age population steadily declined up to 2010 in all major causes of death except suicide. Managers had a higher risk of mortality in all leading causes of death compared with before 1995. Mortality rates among unemployed people steadily decreased for all cancers and ischaemic heart disease. Economic downturn may have caused the prolonged increase in suicide mortality. Unemployed people did not experience any change in mortality due to suicide and cerebrovascular disease and saw a decline in cancer and ischemic heart disease mortality, perhaps because the basic properties of Japan’s social welfare system were maintained even during economic recession.

Economic downturn may affect health outcomes, especially among working-age populations; Japan has been no exception to this^1^. Under the influence of economic changes, the age-standardized mortality rate for the population of working-age males in Japan has undergone major changes, particularly during the late 1990s. The age-standardized mortality rate for those in management and professional roles spiked in 2000, and has remained worse than that of other occupations, including occupations typically associated with a relatively lower socioeconomic status and higher mortality. This increase occurred despite the fact that all occupations are equally treated under the Japanese universal health coverage scheme^2^, and was a unique phenomenon not found in Western countries^3^,^4^. The spike in mortality rates for specific occupations may be associated with particularly harsh economic conditions such as the bursting of the economic “bubble” in the 1990s, also called the “lost decades”, and associated worsening of working conditions^5^.

The Japanese economy again faced serious economic stagnation after the 2008 global financial crisis. The gross domestic product (GDP) growth rate was negative in 2009^6^, and the unemployment rate peaked at 5% in 2009/10, a rate that the Japanese economy has not seen since the period after the Second World War. Many workers experienced job insecurity following a reduction in demand, especially in manufacturing, which was previously Japan’s major industry but was severely affected by a stronger yen and the shift in production to factories outside of Japan^7^. This harsh economic situation changed the working environment and affected workers’ health.

In Japan, the Ministry of Health, Labour and Welfare (MHLW) collects Occupation-specific Vital Statistics^8^ every 5 years, during the same year as the collection of the national population census; the government collects information on occupation as well as cause of death from death certificates submitted to local governments by the families of the deceased. These data can potentially delineate the magnitude of effects on health among working populations by occupation. The present study aimed to determine temporal trends in occupation-specific mortality among Japanese men aged 30 to 59 years in the four leading causes of death for the three decades up to 2010, a period during which Japan faced its worst stagnation since the Second World War. We also aimed to assess the difference in magnitude of changes in mortality associated with this economic downturn by occupation, using a generalized estimating equation model.

## Methods

### Data sources

We obtained individual cause of death data from the Occupation-specific Vital Statistics provided by the Japanese MHLW. We also obtained data on population size by occupation from the national population census, which is implemented at 5-year intervals on 1 October, to calculate occupation-specific death rates. We extracted data for males aged 30–59 years. We excluded those aged 20–29 years, including university and other higher education students, because of the large number of students included in this age band. We also excluded those aged 60 years and over, which was the typical retirement age in Japan during the study period.

### Measurements

Cause of death data were based on information recorded in official death certificates, including the underlying cause of death, completed by physicians based on the sequence of morbid events leading to death, and coded according to the International Classification of Diseases Ninth Revision (ICD-9) (1980–1990) and ICD-10 (1995–2010)^9^,^10^. Some codes were inappropriate for cause-of-death analysis or ill-defined (e.g., heart failure) and were redistributed to be comparable and consistent across data using an algorithm developed by Naghavi and colleagues^11^. Occupations were classified into 10 categories: professional and engineering (hereinafter professional); administrative and managerial including employers (hereinafter management); clerical; sales; services; security services (security); agriculture, forestry, and fisheries (agriculture); transportation and communication (transportation); production and labour work (production/labour); and unemployed. These categories were based on the International Standard Classification of Occupations^12^,^13^. In the years when Occupation-specific Vital Statistics were collected, family members of deceased people were required to select one occupational category from the list of 10 occupations. The list was provided to the family with detailed descriptions and definitions of the occupation categories, as well as job examples for each category.

### Statistical analysis

With data based on 5-year age intervals, we computed an age-standardized mortality rate, directly adjusted to the 1985 Japan standard population^14^, with the same occupation categories as the national census (denominator) and the number of deaths (numerator) for the four leading causes of death: all cancers, suicide, ischaemic heart disease, and cerebrovascular disease. We then categorized occupations into management, professional, unemployed, and other (clerical, sales, services, security, agriculture, transportation and production/labour) based on our analysis of the age-standardized mortality rate trend.

Data were analysed using a generalized estimating equation (GEE) model, assuming a Poisson distribution for the outcome and an exchangeable correlation structure. To model possible changes in mortality in the three specific occupational groups (management, professional and unemployed) in 2000, all models included occupation category as a covariate. A simple step term was included to reflect the potential change across all occupational categories in 2000, and an interaction of the step term with the occupation category was used to identify any additional changes in mortality for these three occupations (management, professional and unemployed). We used backwards stepwise model-building to construct the models. Data were analysed using STATA version 14 (*Stata Statistical Software: Release 14*. College Station, TX: StataCorp LP).

## Results

**Figure 1:**
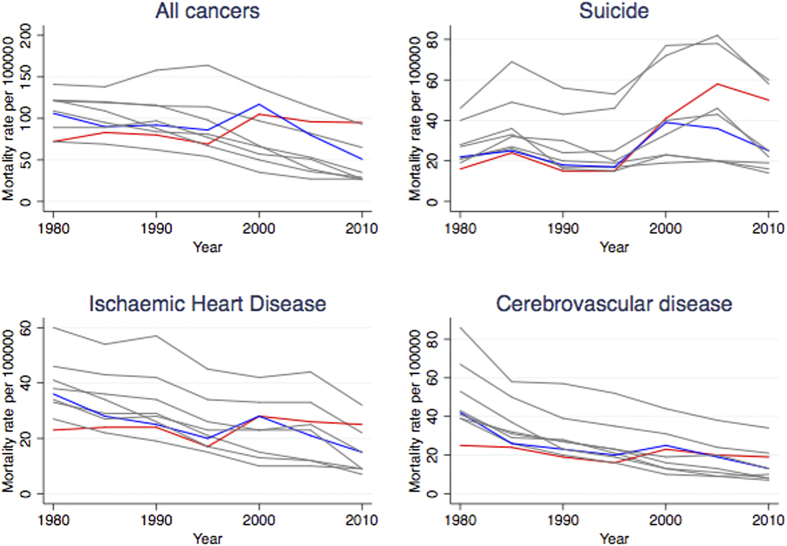
Temporal trends and comparisons of the age-standardized mortality rates (per 100,000) for the four leading causes of death between 1980 and 2010 among Japanese males aged 30–59 years. Red line: managers; Blue line: professionals; Grey lines: Others. See Supplementary Table 1 for detailed data.

Table 1 shows the distribution of occupation categories over time. In 2010, the proportion of those in management roles declined to 2.7%, while the proportion of those unemployed increased to 9.3%. Age-standardized mortality rates for the four leading causes of death declined for all occupations, with the exception of management, where mortality was stagnant (Fig. 1, complete data in Supplementary Table 1).

The GEE model results are summarized in Table 2. For the four leading causes of death, mortality showed a long-term downward trend across the study period, with the exception of suicide. Before 2000, management had lower mortality in all four leading causes of death; however, after 2000, the mortality rate ratio significantly increased in the four leading causes of death (Rate ratio: all cancers 2.29 (95% Confidence Interval (CI): 1.91–2.76, suicide 2.48 (95% CI: 1.83–3.37), ischemic heart disease 2.02 (95% CI: 1.35–3.00), cerebrovascular disease 2.12 (95% CI: 1.38–3.26)). After 2000, the mortality rate for all cancers for those in the professional and unemployed categories also increased compared with other occupations (Rate ratio: professional 1.56 (95% CI: 1.30–1.88), unemployed 1.22 (1.03–1.43)). All occupation categories showed a higher trend in suicide mortality after 2000. Suicide mortality among the unemployed was also high, but did not show a significant change after 2000 (Rate ratio: 0.88 (95% CI: 0.69–1.13).

Occupation-specific relative changes in mortality after 2000 compared with before 2000 are shown in Table 3. Management showed a significant impact on mortality after 2000 in all four causes of death (Rate ratio for all cancers 1.45 (95% CI: 1.18–1.77), suicide 3.47 (95% CI: 2.48–4.84), ischaemic heart disease 1.53 (95% CI: 1.07–2.19) and cerebrovascular disease 1.83 (95% CI: 1.25–2.69)). Suicide mortality significantly increased across all occupation categories after 2000. The unemployed category showed a reduction in all cancers (Rate ratio 0.63 (95% CI: 0.51–0.79)) and ischaemic heart diseases (Rate ratio 0.83 (95% CI: 0.66–1.03)) after 2000.

## Discussion

We examined temporal trends in occupation-specific mortality for the four leading causes of death (all cancers, suicide, ischaemic heart disease, and cerebrovascular diseases) among Japanese males aged 30–59 years from 1980 to 2010. The mortality of those in management roles has remained higher than other occupations since it spiked after 2000, when the Japanese economy stagnated. The mortality rates for all cancers and ischaemic heart disease among unemployed people were not affected by the economic downturn after 2000 and have steadily decreased. Mortality for those in professional roles also showed a downward trend after the spike in 2000, with the exception of suicide mortality. This suggests that economic downturn may have an impact on the mental health of all workers as well as impacting the physical health of managers and employers.

Management is an occupation that has been shown to be associated with better health outcomes in other countries, often due to the better wealth and working conditions of these occupations^15^,^16^. However, this occupation group continues to show higher mortality for Japanese males. A possible reason for the increasing mortality of cardiovascular diseases and cancers previously reported in Japan^17^,^18^ was a greater prevalence of risk factors among managers, but recent reports have shown better risk factor profiles^19^,^20^ or no significant difference in risk factors among managers^21^. Based on the longitudinal study conducted by the MHLW from 2005 to 2010, males aged 50–59 years in management roles had better outcomes for self-rated health and psychological distress compared with other occupations^22^,^23^. These findings may have differed from those of our study because management workers who were experiencing hardship may have dropped out of or not participated in the longitudinal study. Even though most managers, who are often survivors of within-company competition, appear to have better health outcomes, it is necessary to identify and intervene for managers who become vulnerable in terms of their health after confronting business hardship. *Karoshi* (death due to overwork) is one factor that could be associated with the increased mortality for cardiovascular and cerebrovascular diseases, and depression or suicide^24^ through long working hours and job strain^25^. The number of management workers has declined from 8.2% of the workforce in 1980 to 2.7% in 2010, possibly due to organizational restructuring. A previous study conducted a sensitive analysis to assess the impact of this decline, but the results remained unchanged^1^. Future studies should explore the role of managers in more detail, including managers who are employed within companies and those who are themselves employers, as well as the specific industries affected.

Suicide is a priority issue during macroeconomic recession^26^. In Korea, after the 2008 economic recession, managers had a higher relative risk of suicide compared with other occupations^27^. For employers or those who are self-employed, suicide might be attributed to an extreme effort to escape debt. In Japan, after a specified period of holding a relevant policy, insurance companies compensate suicide for the full death benefit^28^. Based on police statistics, approximately 4500 people in Japan committed suicide every year from 2007 to 2009 because of excess debt or business depression, comprising about 15% of the total suicide cases^29^. In our study, we identified a prolonged impact on suicide among specific working populations in Japan. Reducing suicide remains a priority for Japan.

Perceived job insecurity has been recognized as a major determinant of coronary heart disease among workers^30^. The number of workers in production declined from 31.8% in 2005 to 28.1% in 2010, resulting in 1.4 million men aged 30–59 years leaving production work because of the global financial crisis and stronger yen^31^. In the United States from 2008 to 2012, the prevalence of coronary heart disease and stroke was significantly higher among blue collar workers compared with white collar workers aged 55 years or younger, and was possibly related to job insecurity^32^. The mortality rates for ischaemic heart disease and cerebrovascular disease among Japanese men aged 30–59 years appeared stagnant among those in management and production, in contrast to other occupations where mortality decreased (Supplementary Table 1).

Unemployed people are often vulnerable to the poor health effects of economic recession because of government budget austerity in health and social welfare spending during these periods^33^. Other studies have reported that mortality due to stomach cancer^34^ and cerebrovascular diseases^35^ among unemployed people increased, possibly because of reductions in health care expenditure. In contrast, mortality rates for cancers and ischaemic heart disease in unemployed people have steadily reduced, even during Japan’s economic crisis. This may be a result of some austerity measures for health expenditure not being implemented and unemployed people being able to access an equal level of medical services under Japan’s universal health coverage scheme. Unemployed people in Japan could also obtain benefit allowances at around 50 to 80% of their average wage for the 6 months before unemployment for 3–11 months in cases of involuntary termination. These basic properties of Japan’s social welfare system did not change during the 1990s or after 2008, in contrast to countries such as the United Kingdom, where social welfare services were subject to increased austerity after 2008^36^. During economic recession, austerity measures should be discussed not only from the viewpoint of economic sustainability, but should also consider the health of vulnerable populations^37^.

### Limitations

Several limitations of this study mean our results should be interpreted with caution. Since we used the two different national data, there is a possibility of numerator/denominator bias^38^ in this study. However, in a previous analysis of these same two data sets we showed that there is no significant effect of numerator/denominator bias^1^. As family members selected the deceased person’s occupation before submitting the death certificate to the local government, information bias may have occurred through misclassifications in occupational categories. In addition, we did not have detailed information about socioeconomic status such as employment status (regular or uncertain) and education levels. Our statistical analyses modelled the economic crisis as a step term based on previously published work and observed mortality trends. We did not test lag terms or more complex functional forms for the crisis due to the small number of data points, and this small number of data points means that our findings should be interpreted cautiously.

## Conclusion

Economic downturn may have caused a prolonged increase in suicide mortality for Japanese working-age males. Male managers, including employers who faced business hardship, should be targeted for preventive interventions in Japan. Unemployed people enjoyed a reduction in mortality during Japan’s economic downturn, as austerity measures for health and welfare were not implemented. Countries experiencing economic downturn should consider the potential negative impact of austerity measures on the health of the unemployed when crafting responses to crisis, in addition to considering the increased health and mental welfare needs of those retained in the workforce during times of economic shock. This may minimize the impact of economic crises on health and ensure that the social welfare and labour law systems protect workers from harm during times of economic uncertainty.

## Additional Information

**How to cite this article**: Wada, K. and Gilmour, S. Inequality in mortality by occupation related to economic crisis from 1980 to 2010 among working-age Japanese males. *Sci. Rep.* **6**, 22255; doi: 10.1038/srep22255 (2016).

## Supplementary Material

Supplementary Information

## Figures and Tables

**Table 1:** Proportion of Japanese males aged 30–59 by occupation according to the national population census 1980–2010 (%).

**Year**	**1980**	**1985**	**1990**	**1995**	**2000**	**2005**	**2010**
Occupation	n = 24,250,948	n = 25,896,538	n = 25,961,345	n = 26,059,598	n = 25,935,755	n = 25,621,203	n = 24,077,226
Management	8.2	6.3	6.6	6.7	4.1	3.2	2.7
Professional	7.4	9.5	10.6	12.1	13.2	12.6	13.6
Unemployed	4.1	5.3	4.4	5.2	7.0	8.3	9.3
Other	80.3	78.9	78.4	76.0	75.7	75.9	74.4

Other: clerical, sales, services, security, agriculture, transportation, and production/labour.

**Table 2:** Ratios of age-standardized mortality rates for the leading causes of death: Effect of financial crisis (after 2000) by the generalized estimating equation models.

**Cause of death and variable**	**Rate ratio**	**95% Confidence Interval**	**Z-statistic**	**P value**
All cancers				
Time	0.97	0.94–1.00	−1.73	0.08
After 2000	0.63	0.51–0.79	−4.03	<0.001
Occupation				
Management	0.79	0.71–0.89	−4.03	<0.001
Professional	0.97	0.87–1.08	0.48	0.6
Unemployed	5.13	4.69–5.62	35.66	<0.001
Other	ref			
After 2000/Occupation				
After 2000× Management	2.29	1.91–2.76	8.78	<0.001
After 2000× Professional	1.56	1.30–1.88	4.74	<0.001
After 2000× Unemployed	1.22	1.03–1.43	2.31	0.02
After 2000× Other	ref			
Suicide				
Time	0.94	0.90–0.99	−2.26	0.02
After 2000	1.40	1.01–1.93	2.04	0.04
Occupation				
Management	0.62	0.49–0.78	−4.03	<0.001
Professional	0.73	0.58–0.90	−2.85	0.004
Unemployed	7.75	6.55–9.18	23.75	<0.001
Other	ref			
After 2000/Occupation				
After 2000× Management	2.48	1.83–3.37	5.81	<0.001
After 2000× Professional	1.42	1.04–1.93	2.23	0.03
After 2000× Unemployed	0.88	0.69–1.13	−0.97	0.3
After 2000× Other	ref			
Ischaemic heart disease				
Time	0.93	0.88–0.98	−2.63	0.009
After 2000	0.76	0.52–1.10	−1.44	0.1
Occupation				
Management	0.74	0.58–0.95	−2.4	0.02
Professional	0.92	0.73–1.16	−0.74	0.5
Unemployed	7.09	5.93–8.48	21.46	<0.001
Other	ref			
After 2000/Occupation				
After 2000× Management	2.02	1.35–3.00	3.45	0.001
After 2000× Professional	1.32	0.89–1.97	1.35	0.2
After 2000× Unemployed	1.09	0.79–1.50	0.51	0.6
After 2000× Other	ref			
Cerebrovascular disease				
Time	0.84	0.79–0.89	−6.25	<0.001
After 2000	0.86	0.60–1.27	−0.74	0.5
Occupation				
Management	0.61	0.47–0.78	−3.89	<0.001
Professional	0.80	0.64–1.01	−1.85	0.06
Unemployed	5.42	4.57–6.43	19.34	<0.001
Other	ref			
After 2000/Occupation				
After 2000× Management	2.12	1.38–3.26	3.45	0.001
After 2000× Professional	1.47	0.97–2.25	1.81	0.07
After 2000× Unemployed	1.16	0.83–1.62	0.87	0.4
After 2000× Other	ref			

Ref: reference; other occupations: clerical, sales, services, security, agriculture, transportation and production/labour.

**Table 3:** Occupation-specific relative change in mortality after 2000 compared with before 1995.

**Cause of death and occupation group**	**Rate ratio**	**95% Confidence Interval**	**P value**
All cancers			
Management	1.45	1.18–1.77	<0.001
Professional	0.98	0.80–1.21	0.9
Unemployed	0.77	0.66–0.89	<0.001
Other	0.63	0.51–0.79	<0.001
Suicide			
Management	3.47	2.48–4.84	<0.001
Professional	1.99	1.41–2.79	<0.001
Unemployed	1.24	1.01–1.51	0.04
Other	1.40	1.01–1.93	0.04
Ischaemic heart disease			
Management	1.53	1.07–2.19	0.02
Professional	1.00	0.70–1.44	0.99
Unemployed	0.83	0.66–1.03	0.09
Other	0.76	0.52–1.10	0.1
Cerebrovascular disease			
Management	1.83	1.25–2.69	0.002
Professional	1.28	0.88–1.86	0.2
Unemployed	1.00	0.79–1.27	0.98
Other	0.86	0.60–1.27	0.5

Other occupations: clerical, sales, services, security, agriculture, transportation, and production/labour.
