# Supplementary material for: Inequality in mortality by occupation related to economic crisis from 1980 to 2010 among working-age Japanese males
Source: Sci Rep. 2016 Mar 3;6:22255. doi: 10.1038/srep22255 (PMC4776242; doi:10.1038/srep22255)
Supplement: Supplementary Information [file srep22255-s1.doc]

Wada, K. & Gilmour, S. Inequality in mortality by occupation related to economic crisis from 1980 to 2010 among working-age Japanese males

| Supplementary Table. Temporal trends and comparisons of the age-standardized mortality rates (per 100,000) for the leading causes of death between 1980 and 2010 among Japanese males aged at 30–59 years | | | | | | | | |  |
| --- | --- | --- | --- | --- | --- | --- | --- | --- | --- |
|  | | | | | | | | |  |
| a) All cancers | |  |  |  |  |  |  |  |  |
| Occupation | 1980 | 1985 | 1990 | 1995 | 2000 | 2005 | 2010 | †Absolute difference | *%Change |
| All | 144 | 121 | 116 | 107 | 100 | 102 | 77 | −67 | −47 |
| Management | 72 | 83 | 80 | 69 | 105 | 96 | 95 | 23 | 32 |
| Professional | 106 | 90 | 92 | 86 | 117 | 80 | 51 | −55 | −52 |
| Unemployed | 490 | 472 | 557 | 452 | 381 | 338 | 298 | −192 | −39 |
| Other | 105 | 99 | 92 | 88 | 70 | 60 | 33 | −72 | −69 |
| Clerical | 121 | 119 | 116 | 98 | 67 | 39 | 26 | −95 | −79 |
| Sales | 122 | 109 | 88 | 67 | 50 | 36 | 29 | −93 | −76 |
| Services | 141 | 138 | 158 | 164 | 137 | 114 | 93 | −48 | −34 |
| Security | 89 | 89 | 97 | 77 | 57 | 51 | 27 | −62 | −70 |
| Agriculture | 122 | 120 | 115 | 114 | 97 | 82 | 65 | −57 | −47 |
| Transportation | 109 | 95 | 84 | 81 | 66 | 53 | 35 | −74 | −68 |
| Production/labour | 72 | 69 | 62 | 54 | 35 | 27 | 27 | −45 | −63 |
|  |  |  |  |  |  |  |  |  |  |
| b) Suicide |  |  |  |  |  |  |  |  |  |
| Occupation | 1980 | 1985 | 1990 | 1995 | 2000 | 2005 | 2010 | †Absolute difference | *%Change |
| All | 34 | 43 | 31 | 32 | 50 | 53 | 47 | 13 | 38 |
| Management | 16 | 24 | 15 | 15 | 41 | 58 | 50 | 34 | 213 |
| Professional | 22 | 25 | 18 | 17 | 39 | 36 | 25 | 3 | 14 |
| Unemployed | 222 | 238 | 224 | 192 | 244 | 221 | 200 | −22 | −10 |
| Other | 28 | 36 | 24 | 25 | 36 | 39 | 22 | −6 | −21 |
| Clerical | 21 | 27 | 20 | 19 | 23 | 20 | 14 | −7 | −33 |
| Sales | 28 | 36 | 16 | 15 | 23 | 20 | 16 | −12 | −43 |
| Services | 40 | 49 | 43 | 46 | 77 | 78 | 60 | 20 | 50 |
| Security | 19 | 32 | 30 | 20 | 33 | 46 | 22 | 3 | 16 |
| Agriculture | 46 | 69 | 56 | 53 | 72 | 82 | 58 | 12 | 26 |
| Transportation | 27 | 33 | 24 | 25 | 40 | 43 | 25 | −2 | −7 |
| Production/labour | 21 | 26 | 17 | 17 | 19 | 20 | 19 | −2 | −10 |
|  |  |  |  |  |  |  |  |  |  |
| c) Ischaemic heart disease | | |  |  |  |  |  |  |  |
| Occupation | 1980 | 1985 | 1990 | 1995 | 2000 | 2005 | 2010 | †Absolute difference | *%Change |
| All | 44 | 40 | 38 | 31 | 30 | 32 | 27 | −17 | −39 |
| Management | 23 | 24 | 24 | 17 | 28 | 26 | 25 | 2 | 9 |
| Professional | 36 | 28 | 25 | 20 | 28 | 21 | 15 | −21 | −58 |
| Unemployed | 218 | 201 | 253 | 172 | 148 | 147 | 114 | −104 | −48 |
| Other | 36 | 31 | 28 | 24 | 20 | 22 | 11 | −25 | −69 |
| Clerical | 33 | 29 | 29 | 21 | 15 | 12 | 7 | −26 | −79 |
| Sales | 41 | 34 | 26 | 17 | 13 | 12 | 9 | −32 | −78 |
| Services | 60 | 54 | 57 | 45 | 42 | 44 | 32 | −28 | −47 |
| Security | 38 | 36 | 34 | 26 | 23 | 23 | 9 | −29 | −76 |
| Agriculture | 46 | 43 | 42 | 34 | 33 | 33 | 22 | −24 | −52 |
| Transportation | 34 | 27 | 28 | 23 | 23 | 25 | 15 | −19 | −56 |
| Production/labour | 27 | 22 | 19 | 15 | 10 | 10 | 9 | −18 | −67 |
|  |  |  |  |  |  |  |  |  |  |
| d) Cerebrovascular disease | | |  |  |  |  |  |  |  |
| Occupation | 1980 | 1985 | 1990 | 1995 | 2000 | 2005 | 2010 | †Absolute difference | *%Change |
| All | 58 | 42 | 34 | 30 | 27 | 25 | 21 | −37 | −64 |
| Management | 25 | 24 | 19 | 16 | 23 | 20 | 19 | −6 | −24 |
| Professional | 42 | 26 | 23 | 20 | 25 | 19 | 13 | −29 | −69 |
| Unemployed | 252 | 175 | 167 | 154 | 115 | 103 | 84 | −168 | −67 |
| Other | 51 | 35 | 28 | 24 | 19 | 18 | 11 | −40 | −78 |
| Clerical | 43 | 29 | 28 | 21 | 13 | 9 | 7 | −36 | −84 |
| Sales | 53 | 37 | 23 | 19 | 13 | 11 | 8 | −45 | −85 |
| Services | 86 | 58 | 57 | 52 | 44 | 38 | 34 | −52 | −60 |
| Security | 41 | 31 | 27 | 23 | 16 | 13 | 8 | −33 | −80 |
| Agriculture | 67 | 50 | 39 | 35 | 31 | 24 | 21 | −46 | −69 |
| Transportation | 39 | 32 | 27 | 23 | 19 | 20 | 13 | −26 | −67 |
| Production/labour | 39 | 26 | 20 | 16 | 10 | 9 | 10 | −29 | −74 |
|  |  |  |  |  |  |  |  |  |  |
| †Absolute difference in the age-standardized mortality rate per 100,000 between 1980 and 2005 | | | | | | | |  |  |
| *Difference between 1980 and 2010 rates expressed as the percentage of the 1980 rate | | | | | | | |  |  |
